# Anchored Oxygen‐Release‐Suppression Phase Stabilizing High‐Voltage Ni‐Rich Cathode Materials

**DOI:** 10.1002/anie.9916183

**Published:** 2026-08-23

**Authors:** Ziqi Liu, Yiming Zhang, Yong Chen, Meng Yao, Yangyang Wang, Du Yuan, Haitao Zhang, Yun Zhang, Guoxiu Wang

**Affiliations:** ^1^ College of Materials Science and Engineering Sichuan University Chengdu China; ^2^ Centre For Clean Energy Technology University of Technology Sydney Sydney New South Wales Australia; ^3^ College of Materials Science and Engineering Changsha University of Science and Technology Changsha Hunan China; ^4^ Institute of Process Engineering Chinese Academy of Science Beijing China

**Keywords:** anchored strategy, dual architecture, Ni‐rich cathode, oxygen emission, structural stability

## Abstract

The Ni‐rich LiNi_x_Co_y_Mn_1−x−y_O_2_ (x ≥ 0.9) cathode materials (NCMs) have been considered promising for next‐generation Li‐ion batteries owing to their high‐energy density. However, their practical application is hindered by gas evolution and rapid capacity degradation, primarily caused by irreversible oxygen release and structural instability. Herein, a facile one‐step anchoring strategy is proposed to overcome this challenge by engineering a precisely tailored dual‐architecture LiNi_0.9_Co_0.05_Mn_0.05_O_2_ (DA‐NCM). This strategy constructs a dual‐phase surface architecture on Ni‐rich cathodes, where perovskite‐phase La_4_LiNiO_8_ mitigates surface oxygen instability and interfacial degradation, and inert La_2_Mo_2_O_9_ anchors the lattice to avoid the detachment of La_4_LiNiO_8_ during cycling via enhanced La─O bond pinning effect. This dual‐architecture design provides a robust strategy for stabilizing the interface, surface, and bulk phase of Ni‐rich cathodes, enabling durable oxygen regulation, suppressed structural degradation, and stable high‐voltage operation under demanding cycling conditions. Thus, our DA‐NCM cathodes demonstrate excellent capacity retention of 95.7% at 4.3 V and 93.6% at 4.5 V after 200 cycles and remarkable stability even at an elevated temperature (50°C) and high voltage (4.5 V), confirming their markedly enhanced electrochemical stability. This precision design of dual architecture provides a new pathway for developing high‐energy‐density cathode materials with long cycle life.

## Graphical abstract

A one‐step dual‐structure strategy was proposed to synthesize LiNi_0.9_Co_0.05_Mn_0.05_O_2_ (DA‐NCM) material, which is rich in oxygen vacancies and coated with a perovskite‐type La_4_LiNiO_8_ layer stabilized by La─O anchoring provided by La_2_Mo_2_O_9_. This strategy effectively suppressed structural degradation and oxygen release, thereby achieving stable high‐voltage cycling performance.

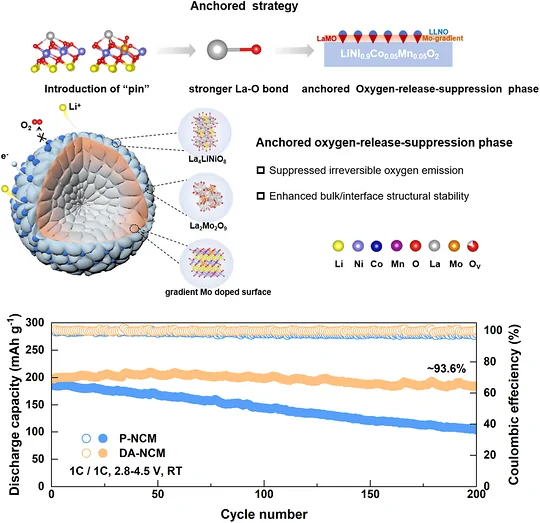

## 1 Introduction

The widespread adoption of electric vehicles (EVs) has accelerated the demand for next‐generation lithium‐ion batteries (LIBs) with ultrahigh energy density and long cycle life [1, 2]. Layered oxide cathodes (LiNi_x_Co_y_Mn_1‐x‐y_O_2_) currently occupy about 50%–60% of the EV battery market, and battery manufacturers are increasingly considering adopting cathodes with ultra‐high nickel content (90%–100%) to achieve superior energy densities. Ni‐rich LiNi_0.9_Co_0.05_Mn_0.05_O_2_ (NCM) cathodes are promising candidates for next‐generation LIBs due to their high theoretical specific capacity (>200 mAh g^−1^), elevated operating voltage (3.6–3.8 V vs. Li^+^/Li), and high theoretical energy density exceeding 800 Wh kg^−1^ [3, 4]. Unfortunately, the increased nickel content and higher operating voltages facilitate charge transfer from oxygen 2p to transition‐metal (TM) 3d orbitals, inducing detrimental oxygen evolution and undermining cathode stability [5]. Furthermore, highly reactive oxygen species on the cathode surface readily react with organic electrolyte, resulting in oxygen loss, phase transitions of the cathode material, and the formation of a thick and heterogeneous cathode‐electrolyte interphase (CEI), which significantly impedes Li^+^ ion diffusion kinetics [6, 7]. Additionally, the pronounced contraction of the lattice c‐axis upon deep delithiation undermines the mechanical integrity of NCM particles, inducing the formation of extensive microcracks [8]. These microstructural defects further accelerate gas release and structural degradation [9, 10, 11]. Moreover, such issues are exacerbated under elevated operating temperatures. As a result, these challenges have seriously hindered the large‐scale implementation of Ni‐rich NCM cathodes.

Numerous strategies, such as surface engineering and bulk phase engineering, have been explored to address the critical challenges of irreversible oxygen release and detrimental structural evolution in Ni‐rich NCM cathodes [12]. Surface engineering with inert oxide coatings or Li^+^ ion‐conductive coatings has been widely used to suppress surface side reactions and enhance mechanical stability. Nevertheless, these approaches always suffer from limited long‐term effectiveness and insufficient Li^+^ mitigation under high‐voltage or high‐temperature operating conditions [13, 14]. During cycling, conventional coatings tend to delaminate from the bulk cathode material due to weak interfacial adhesion, significant lattice mismatch, and pronounced anisotropic lattice contraction along the c‐axis [15, 16]. La‐based materials are widely used as coating materials that can effectively enhance the electrochemical performance of Ni‐rich cathodes. However, although inert La‐based coatings (e.g., La_2_Mo_2_O_9_) improve cycling durability, they may also slow cathode–electrolyte interfacial kinetics and require additional high‐temperature processing [17]. La‐based lithium‐ion conductor coatings (e. g., LiLaO_2_) can effectively promote lithium‐ion transport; however, they fail to effectively mitigate the oxygen evolution issue associated with high‐nickel cathodes, offer limited improvement in the safety performance of Ni‐rich cathodes, and cannot induce the formation of an inorganic‐rich CEI [18]. Moreover, the introduction of heterogeneous or inert coatings itself can lead to a decline in the cathode's kinetic performance and result in a reduction in capacity [19]. In addition, conventional coatings rarely regulate the morphology or microscopic structural evolution of cathode particles, and they generally fail to stabilize lattice oxygen or provide oxygen‐storage functionality. Alternatively, bulk‐phase elemental doping can facilitate Li^+^ transport, suppress cationic mixing, and strengthen the TM─O bond [20, 21, 22], but dopant atoms are typically inert and tend to occupy lithium or transition metal sites, excessive doping would reduce reversible capacity. Furthermore, due to the weak suppression of surface/interface side reactions via doping, constructing a robust and stable CEI remains challenging [23, 24, 25]. Introducing a strain‐retardant phase into NCM has been demonstrated to effectively mitigate the anisotropic lattice strain in high‐nickel cathodes in recent study [26]. However, the introduction of a heterogeneous phase reduces capacity and delivers only limited improvements in surface stability [19]. Although dual modification can integrate the benefits of doping and coating, its practical implementation remains challenging [27, 28, 29, 30]. Multistep reactions complicate synthesis [28], increase the risk of air exposure and residual‐lithium formation [5]. Coordinated component synergy remains difficult to achieve and may be weakened by mutual interference, mainly because multistep synthesis increases process complexity and the mechanism of cooperative stabilization is still poorly understood [31]. Therefore, despite numerous state‐of‐the‐art surface and bulk engineering, effective integration is challenging and may even undermine performance. A deeper mechanistic understanding of multi‐component modifications is critical to develop a simple strategy for achieving structural and interfacial stability alongside robust long‐term cycling.

Herein, we report a one‐step anchoring strategy to build a dual‐architecture design of NCM (DA‐NCM), enabling simultaneous structural anchoring and oxygen‐suppressing in Ni‐rich NCM cathodes, thus improving interfacial and structural stability without capacity loss. Using a facile one‐step synthesis method, a dual‐architecture modification strategy was developed to construct a La_4_LiNiO_8_ (LLNO) primary phase, anchoring it with co‐generated oxygen‐defective La_2_Mo_2_O_9_ (LaMO) phase, and introducing Mo doping within DA‐NCM. Through density functional theory (DFT) calculations, the positional tendency of La/Mo during one‐step synthesis and a reduced Li^+^ ion transport barrier have been determined in DA‐NCM. Both LLNO and inert LaMO phases function as oxygen‐release‐suppression components, modulating oxygen reactivity and enhancing the material's lattice oxygen stability. This dual‐architecture design effectively suppresses gas evolution during operation, as evidenced by in situ differential electrochemical mass spectrometry (DEMS) tests. The strong covalent La─O bond between LLNO and NCM realizes a tight anchoring of the LLNO phase, which could inhibit the harmful evolution of the bulk/interfacial structure during cycling and form a stable CEI simultaneously. Additionally, we demonstrate that controlled interfacial modification alleviates the initial capacity loss caused by lattice mismatch in heterogeneous coatings. Instead, it significantly enhances discharge capacity by improving kinetic properties. Thus, this anchored oxygen‐release‐suppression strategy could inhibit structural degradation, mitigate oxygen emission, and regulate lattice breathing in LiNi_0.9_Co_0.05_Mn_0.05_O_2_, resulting in superior cyclability of DA‐NCM at a high operating voltage and elevated operating temperatures. This work provides a new perspective that not only integrates multiple strategies for stabilizing Ni‐rich NCM cathodes but also reveals their synergistic interactions, offering new design principles for highly stable NCM cathodes.

## 2 Results and Discussions

### 2.1 Design of Dual‐Architecture NCM With an Anchored Oxygen‐Release‐Suppression Phase Strategy

Figure 1a illustrates the anchoring of the LLNO phase and the pinning role of the LaMO phase in DA‐NCM materials. The dual‐architecture coating was designed through DFT‐guided calculations and preliminary experiments. First, to better understand the mechanism of La/Mo interactions in the DA‐NCM and the spatial distribution of LaMO and LLNO, we performed DFT calculations to evaluate the energy of the doped La and Mo on different layers of NCM. As shown in Figure 1b–d, La exhibits the lowest energy on the external surface, indicating its stabilization in the outer layer. While Mo exhibits the highest energy on the external surface, which is unstable. Although Mo is more inclined to exert a bulk doping effect, due to its positive average segregation energy (Es) [32], it tends to aggregate at grain boundaries and grain surfaces. In addition, electrostatic repulsion with Ni further hinders the incorporation of Mo into the bulk lattice [33]. As a result, Mo would not uniformly dope into the bulk lattice but preferentially segregate at the material surface, resulting in trace amounts of near‐surface doping. Consequently, this distribution establishes a radial Mo gradient within the DA‐NCM secondary particles, with the Mo concentration decreasing from the outer region toward the core, as depicted in Figure 1e. It enables a dual‐architecture strategy featuring an outer layer of LLNO and an inner layer of LaMO. The results of the preliminary experiment confirmed that an optimized La/Mo ratio and sintering process could not only form LLNO [9] but also ensure the generation of a pure LaMO‐anchored phase with high oxygen‐ion conductivity and abundant oxygen vacancies [34], which is proved by x‐ray diffraction (XRD) and high‐resolution transmission electron microscopy (HRTEM) images [35]. As shown in Figure S1a, the XRD pattern matches that of standard LaMO patterns, and the TEM image (Figure S1b) reveals a crystal plane with a spacing of 0.36 nm, pointing to the (200) crystal plane of LaMO. Selected area electron diffraction (SAED) analysis of pure‐phase LaMO powder further confirms its crystal structure as shown in Figure S1c. Results of Raman spectroscopy and electron paramagnetic resonance (EPR) spectroscopy also corroborate this conclusion (Figures S2 and S3). Guided by the above preliminary experiments and calculations, we devised the dual‐architecture anchoring strategy, wherein oxygen‐release‐suppression LLNO outer layer is anchored to the NCM surface via the inert LaMO inner structure. Additionally, the excellent lithium‐ion conductivity provided by the LLNO main coating and Mo doping will also be an advantage of the dual‐architecture strategy (Figure 1c,f).

**FIGURE 1:**
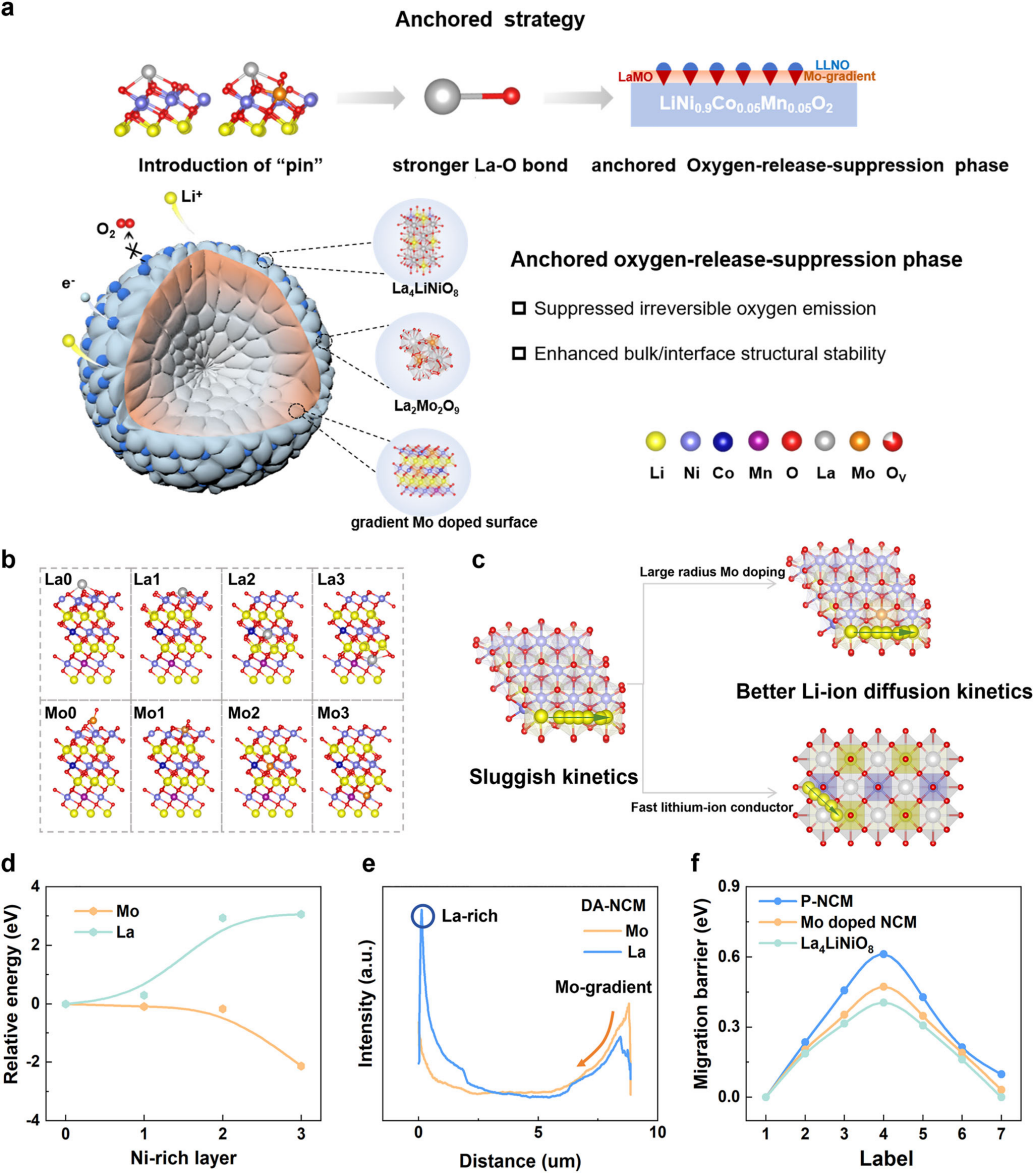
DFT calculations assist in anchored strategy design. (a) Schematic illustrations of the anchored dual architecture strategy. (b) Structures of La and Mo doping on the surface, first, second, and third layer of NCM. (c) Theoretical models for Li^+^ diffusion paths (d) The relative energy of La and Mo in different layers. (e) The distribution of La and Mo in DA‐NCM cross sections. (f) Li^+^ diffusion energy barriers of NCM, Mo‐doped NCM, and La_4_LiNiO_8_ phase.

To evaluate the impact of the dual‐architecture anchoring strategy on the cathode structure, the detailed structures of the pristine NCM (P‐NCM), La‐coated NCM (La‐NCM, coated only with LLNO), and DA‐NCM were systematically characterized by x‐ray diffraction (XRD) and neutron powder diffraction (NPD). As shown in Figures 2a and S4, all well‐defined strong peaks are consistent with the *α*‐NaFeO_2_ hexagonal layered structure (R‐3m), and the weak diffraction peaks of DA‐NCM between 24° and 36° are in accordance with the LLNO phase. In addition, there is little effect on the (003) peak position in La‐NCM compared with P‐NCM, but it shifts to a lower angle in DA‐NCM. These results indicate that La preferentially constructs surface coatings, whereas Mo exhibits a doping effect in DA‐NCM, in agreement with the DFT theoretical calculations. These effects lead to an increase in lattice parameters [36]. The GSAS II software was used to obtain the lattice parameters of P‐NCM, La‐NCM and DA‐NCM via the Rietveld refinement method (Figure S5 and Tables S1–S4). We found that the Ni^2+^ occupancy in the Li^+^ site decreases from 3.36% (P‐NCM) to 2.41% (La‐NCM) and 1.66% (DA‐NCM), which has also been confirmed by the results of NPD (Figures 2b and S6; Tables S5–S8). The XRD data of other samples (Figure S7 and Table S9) with different modification ratios also support the above conclusion. The x‐ray absorption near edge structure (XANES) measurement shows that the Ni K‐edge of DA‐NCM and P‐NCM materials overlap well (Figure S8). To further investigate the influence of dual‐architectural strategies on the surface and bulk chemical properties of NCM, we conducted soft x‐ray absorption spectroscopy (sXAS) measurements using both surface‐sensitive total electron yield (TEY) and total fluorescence yield (TFY) channels. The peak intensity ratio between the high energy shoulder (L_3high_) and low energy shoulder (L_3low_) of Ni L_3_‐edge is positively correlated to the Ni oxidation state. As shown in Figure 2c, L_3high_/L_3low_ of DA‐NCM in TEY mode is markedly higher than that in P‐NCM, indicating higher surface Ni oxidation states which is consistent with the analysis of Ni valence states on the surface in x‐ray photoelectron spectroscopy (XPS) testing (Figure 2d, Figure S9a and Table S10). In TFY mode, DA‐NCM also exhibits a slightly higher ratio of L_3high_/L_3low_, indicating an ordered structure with less Li/Ni mixing in the bulk phase of DA‐NCM. The results indicate that although the dual‐architecture strategy may induce Mo doping effects, it does not enhance Li/Ni mixing in DA‐NCM, Instead, DA‐NCM presents a more ordered structure, which may be related to the special enhanced TM─O bonding and fewer bulk lattice oxygen vacancies [37].

**FIGURE 2:**
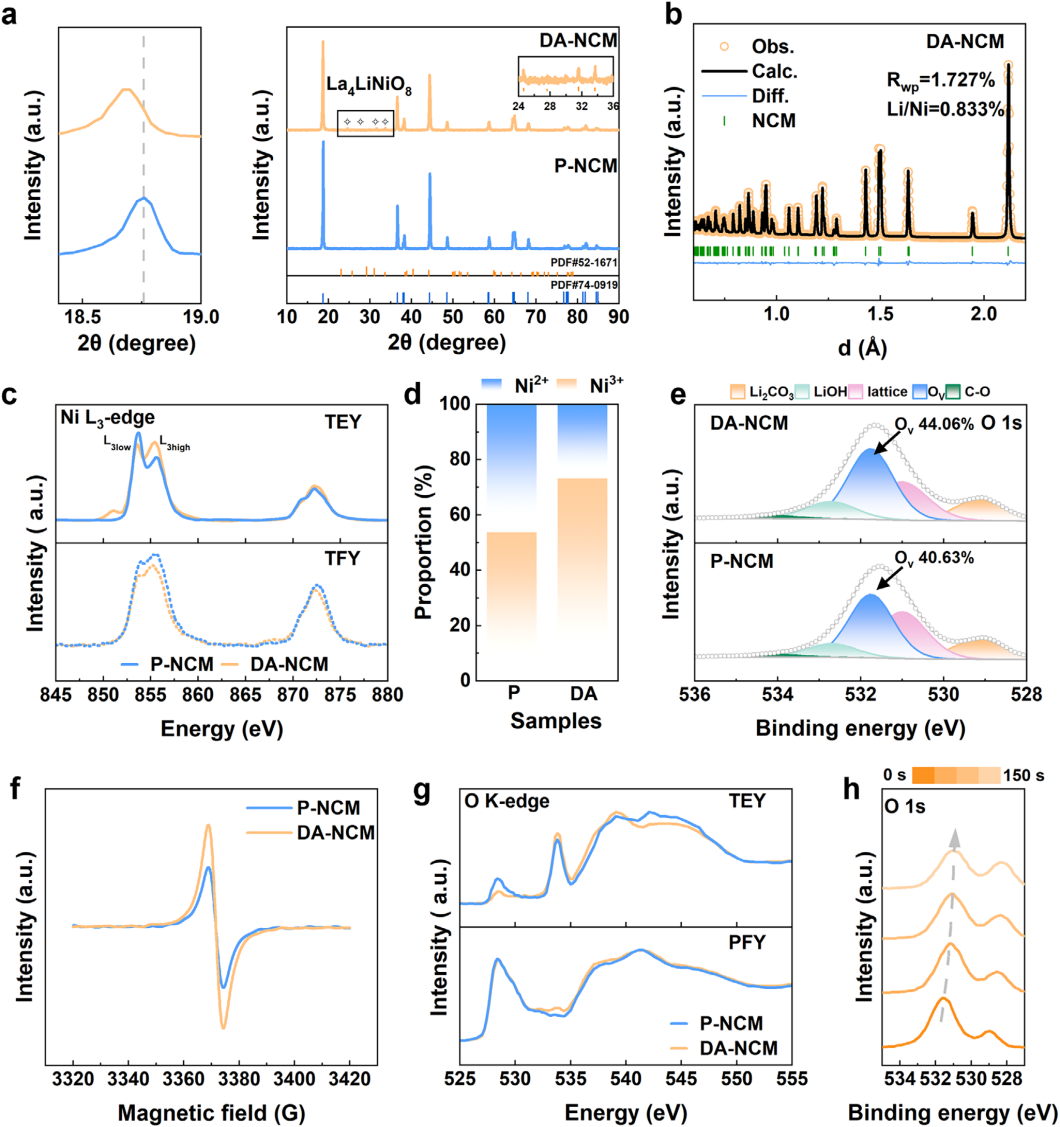
Crystal structure and surface chemistry analysis. (a) XRD patterns of P‐NCM, DA‐NCM and the magnified patterns of (003) peak. (b) NPD Rietveld refinement of DA‐NCM. (c) sXAS spectra of Ni L_3_‐edge of P‐NCM and DA‐NCM. (d) Proportion of Ni^2+^ and Ni^3+^ according to XPS spectra of Ni 2p. (e) XPS spectra of O 1s. (f) EPR results of P‐NCM, DA‐NCM. (g) sXAS spectra of O K‐edge of P‐NCM and DA‐NCM. (h) XPS depth profiles of O 1s of DA‐NCM as a function of Ar etching time from 0 s to 150 s.

The surface characteristics of the cathode materials were thoroughly investigated. The XPS spectra of O 1s for all the samples indicates that the surface oxygen vacancy concentration of DA‐NCM is higher than that of P‐NCM but lower than La‐NCM (Figures 2e, S9b, and Table S11). The XPS spectra of Mo 3d and La 3d confirm the existence of LLNO and LaMO on the surface of DA‐NCM (Figure S9c,d), and the valence states of other elements remained stable after the modification. (Figure S9e, f). Through the Ar‐etched XPS test for DA‐NCM particles, the gradual decrease Mo content from the surface toward the interior is confirmed (Figure S10). It can also be seen that the O 1s etching spectra (Figure 2h) shift towards higher binding energy at the surface, suggesting stronger O bonds with neighboring cations in DA‐NCM particles. Notably, the shake‐up satellite peak to parent peak intensity ratio of La 3d_5/2_ (I_S_/I_M_, covalent factor) of DA‐NCM is higher than that of La‐NCM (Figure S11), and the characteristic peak shifts towards high binding energy, suggesting the strongest La─O covalent bonds [38]. Therefore, the LLNO oxygen‐release‐suppression phase would be more firmly anchored onto the DA‐NCM surface.

Further investigation of oxygen vacancy characteristics across the bulk and surface was conducted by electron paramagnetic resonance (EPR) spectroscopy. The overall oxygen vacancy concentration [39] of DA‐NCM is higher than that of P‐NCM according to the EPR results (Figure 2f), and slightly lower than that of La‐NCM (Figure S12), consistent with the XPS results. In Ni‐rich cathodes, an inverse thermodynamic relationship exists between lattice oxygen stability and cation mixing, whereby cation mixing destabilizes lattice oxygen, facilitating oxygen loss and the formation of oxygen vacancies, which in turn further promote cation mixing in a self‐reinforcing cycle [40]. Therefore, it indicates that DA‐NCM forms an extra oxygen‐vacancy‐rich layer (LaMO) with a stable and high concentration of oxygen vacancies confined to the surface, accompanied by suppressed diffusion of oxygen vacancies into the bulk during the sintering process [41]. Consequently, surface Mo doping is not accompanied by increased Li/Ni mixing. The above conclusion is also confirmed by the distribution of Ni elements in the cross‐section of the Ni‐rich cathode. As shown in Figure S13, the Ni element in P‐NCMs with higher harmful intrinsic oxygen vacancies stabilizes later because positively charged oxygen vacancies and Ni^2+/3+^ metal ions mutually repel each other [42]. O K‐edge sXAS spectra under TEY mode exhibits a reduction in the pre‐edge peak at 529 eV of DA‐NCM, which correlates with TM 3d‐O 2p hybridization [43], demonstrating more oxygen vacancy on the surface of DA‐NCM caused by the oxygen‐ vacancy‐rich LLNO and LaMO (Figure 2g).

The microstructures of all the as‐prepared samples were characterized by scanning electron microscopy (SEM), transmission electron microscopy (TEM), and special aberration‐corrected scanning transmission electron microscopy (AC‐STEM). The nano‐island coating is deposited on the surface of DA‐NCM and other modified samples (Figures S14–S16). Focused ion‐beam cut cross‐sectional scanning electron microscopy (FIB‐SEM) images show refined primary grains and smaller‐sized secondary particles in DA‐NCM (Figures 3a and S17), which is effective in mitigating strain accumulation during cycling. The increased Brunauer‐Emmett‐Teller (BET) surface area of the DA‐NCM sample confirms the refinement of particle size (Figure S18 and Table S12). AC‐STEM (Figure 3b), high‐resolution TEM image (Figure 3c) and the corresponding SAED patterns of Figure 3c (Figure S19) confirm the formation of LLNO nanoparticles on the surface of bulk NCM of DA‐NCM (Figure S20). Additionally, an LaMO layer belonging to the P213 space group exists on the DA‐NCM surface as well, as demonstrated by fast Fourier transform (FFT) analysis (Figure 3d).

**FIGURE 3:**
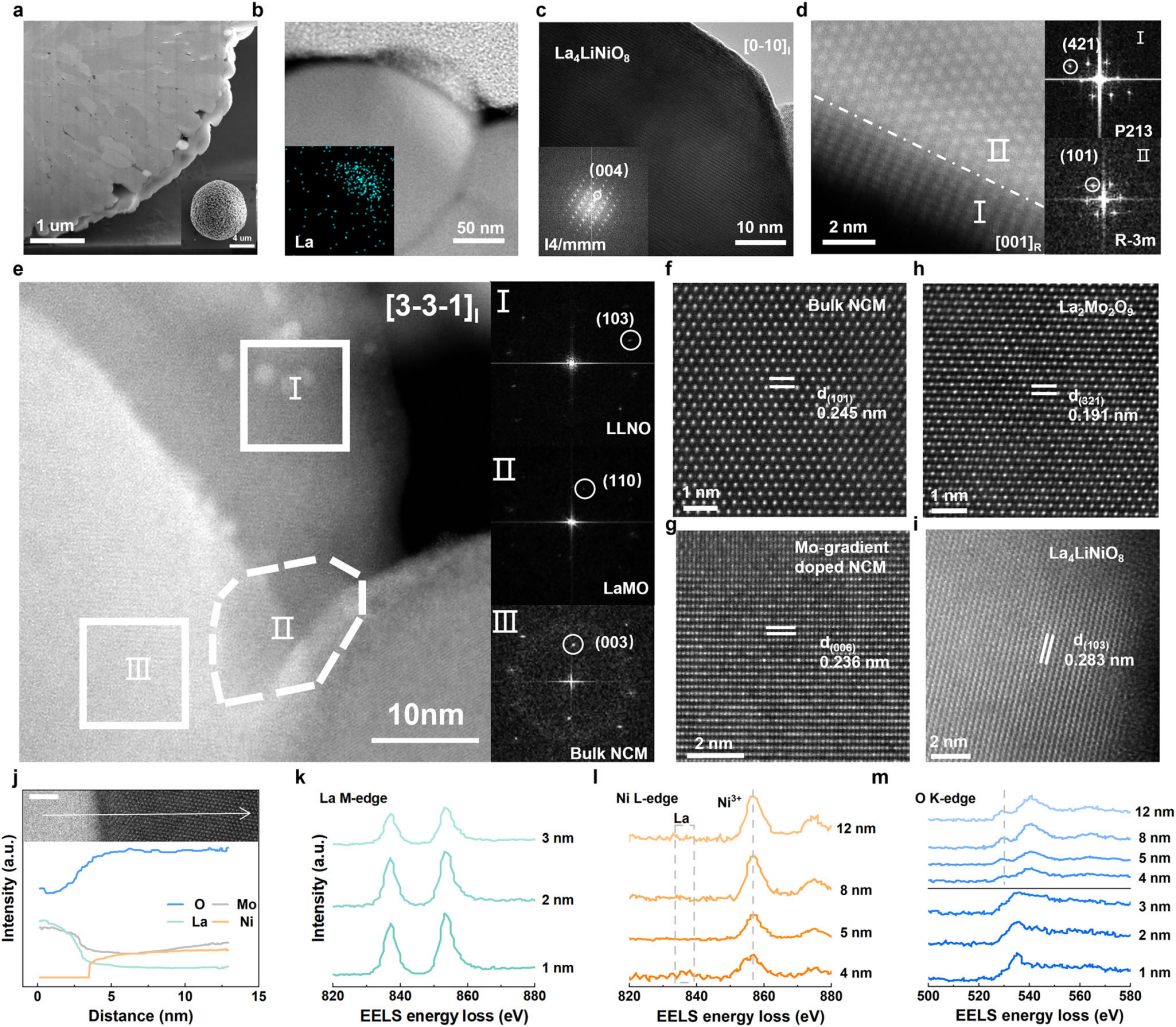
Anchored dual‐architecture precision design. (a) Cross‐sectional FIB‐SEM images of DA‐NCM. (b) HAADF‐STEM image of La‐rich nanoparticles on the surface of DA‐NCM. (c) HRTEM image of LLNO structure. (d) HAADF‐STEM image of LaMO “pin” structure on the surface of DA‐NCM. (e) HAADF‐STEM image of the three‐phases junction of LLNO, LaMO, and NCM and the corresponding FFT patterns for different regions. (f‐i) Atomic resolution images of bulk NCM (f), Mo‐doped NCM (g), LaMO (h), and LLNO (i) of DA‐NCM (j) EELS line scan results along the white arrow, progressively scanning from the 3 nm heterogeneous coating on the DA‐NCM surface toward the bulk phase of NCM. Scale: 2 nm. (k–m) EELS line analysis of La M‐edge and Ni L‐edge (k, l) and O K‐edge (m) at different depths along a white arrow in panel j.

To further clarify the phase distribution of NCM, LLNO, and LaMO and to demonstrate the anchoring structure, STEM images of the connection region between the three phases are presented. As shown in Figures 3e and S21, FFT analysis demonstrates the primary component of the surface nanocoating is LLNO (Region I), and the diffraction spots corresponding to its (103) crystal plane can be identified, with a crystal plane spacing of 0.284 nm. A phase distinct from both LLNO and NCM appears beneath LLNO at the interphase between LLNO and NCM (region III). It is identified as LaMO (region II). The diffraction spots correspond to its (110) crystal plane, with a crystal plane spacing of 0.506 nm. Additionally, the DA‐NCM surface in region III shows a larger (003) interplanar spacing than bulk NCM, indicating Mo doping near the NCM surface. It is also noticed that the (110) crystal plane of LaMO exhibits a minor orientation difference and a similar interplanar spacing compared to the (003) crystal plane of NCM, which may help mitigate the lattice mismatch issue during the lattice breathing between the heterogenous coating and NCM [44]. Figure 3f–i shows atomic‐resolution images of LLNO, LaMO, NCM, and Mo‐doped NCM within DA‐NCM. This anchoring configuration is absent in La‐NCM (Figure S22).

Line scanning electron energy loss spectra (EELS) at the surface along with the guideline of DA‐NCM shows that the heterogeneous phase (∼3 nm) closely linked to the NCM does not contain Ni elements, only La, Mo, and O elements are present. Moreover, the distribution of La and Mo in this heterogeneous coating is synchronized. (Figure 3j). EELS results of different elements at different depths show that La is enriched in the ∼3 nm thick surface phase, while it is not visible in the main NCM phase, as shown in Figure 3k. In contrast, Ni is not present in this heterogeneous coating but exists solely in the bulk NCM phase (4‐12 nm). Moreover, no significant chemical shift is observed for the Ni L‐edge at different depths (Figure 3l). Such results further demonstrate that another phase, distinct from LLNO, has formed on the NCM surface. Meanwhile, it is noteworthy that the O K‐edge exhibits distinct characteristics in the surface heterogeneous coating (1‐3 nm) and the bulk NCM phase (4‐12 nm). The pre‐edge peak at the surface corresponding to the hole‐type electronic state of the O atomic orbital disappears, signaling the presence of abundant oxygen vacancies in this heterogeneous structure [45]. While in the main phase NCM, the pre‐edge peak at ∼530 eV is present and shifts to a higher EELS energy loss at the surface, indicating stronger O‐bonding with neighboring cations (Figure 3m). The above results further confirm the existence of the LaMO structure with abundant oxygen vacancies on the surface of DA‐NCM. This oxygen‐vacancy‐rich LaMO structure is the crucial structure that anchors the LLNO oxygen‐release‐suppression phase on the surface of the NCM while enhancing structural stability and regulating oxygen activity.

### 2.2 Superior Electrochemical Performance of DA‐NCM

Electrochemical measurements were carried out on all samples to systematically evaluate their electrochemical performance under room temperature conditions (RT). As shown in Figures 4a and S23a,b, DA‐NCM exhibits the highest initial Coulombic efficiency. There is no capacity penalty upon introducing the inert phase, attributable to microstructural refinement and enhanced ionic conductivities from LLNO phases. Measurements of the thermodynamic equilibrium potential confirm consistency between measured capacity and state of charge (Figure S24). The initial cycle's differential capacity (dQ/dV) curves reveal that the H2‐H3 redox peak of DA‐NCM exhibits reduced intensity relative to P‐NCM (Figure S25), indicating the suppressed phase transition of DA‐NCM [46]. As shown in Figures S26 and 4b, the cycle stability of the DA‐NCM has been substantially improved. The capacity retention of DA‐NCM is 95.7% and 93.6% after 200 cycles of 1 C at 4.3 and 4.5 V, which is the best cycling performance among all samples (Figure S23c,d) and ranks one of the best compared with the recently reported studies (Table S13). The high‐voltage cycling stability described above is maintained even under high‐load conditions (Figure S27a). Furthermore, when DA‐NCM was assembled into a full cell with a graphite anode, it exhibited no significant capacity decay after 100 cycles at 4.2 V, and its cycling performance remained superior to that of P‐NCM even at the high voltage of 4.5 V (Figure S27b).

**FIGURE 4:**
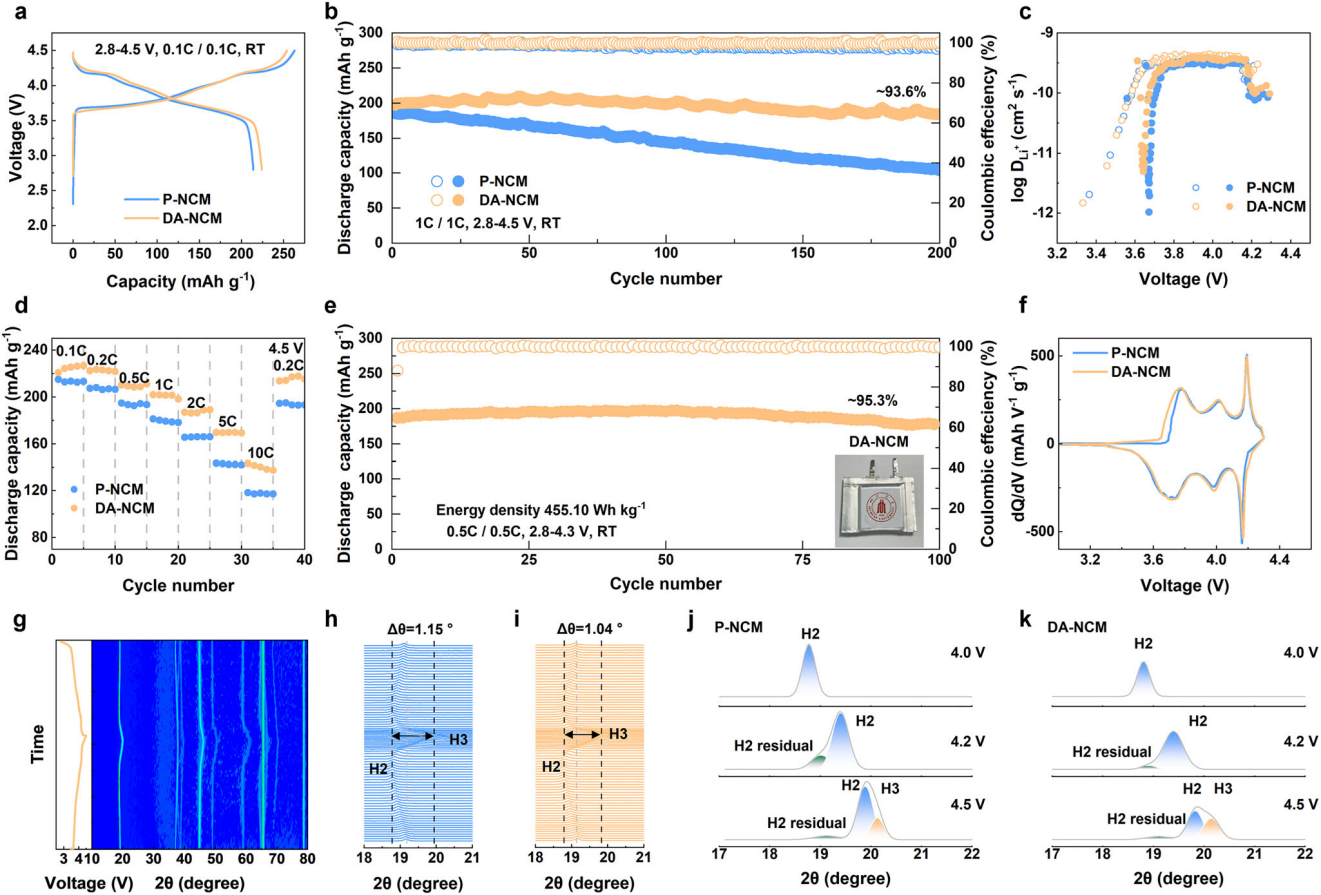
Enhanced electrochemical performance brought by the anchored strategy. (a) The first cycle charge/discharge profiles under 2.8‐4.5 V at 0.1 C. (b) Cycling performances of P‐NCM and DA‐NCM under 2.8‐4.5 V at 1 C, RT. (c) Lithium‐ion diffusion coefficient of P‐NCM and DA‐NCM. (d) Rate capability under 2.8‐4.5 V. (e) Electrochemical performance of pouch cell for DA‐NCM. (f) GITT derived dQ/dV curves of P‐NCM and DA‐NCM. (g) Full range contour plot of in‐situ XRD data on DA‐NCM during the first cycle. The left curve shows the corresponding charge‐discharge profile. (h, i) Enlarged (003) specific reflections from in situ XRD patterns of P‐NCM (h) and DA‐NCM (i). (j, k) (003) profiles and their deconvolutions at different voltages in the in situ XRD test results of fresh P‐NCM (j) and DA‐NCM (k) cathodes.

DA‐NCM further demonstrates excellent high‐rate performance and capacity recovery. The electrode delivered a capacity of 140.4 mAh g^−1^ even at 10 C, which is much higher than that of P‐NCM (118.4 mAh g^−1^) (Figures 4d and S28). This outstanding performance could be attributed to the enhanced Li^+^ transport dynamics (Figure S29, the mass loadings of cathodes are listed in Table S14) and decreased surface charge transfer resistance (R_ct_) of DA‐NCM (Figure S30) due to the reduced degree of Li/Ni mixing, refined particles, and excellent ionic conductivity of LLNO coating. The pouch cell assembled with DA‐NCM cathode maintains a capacity retention rate of 95.3% after 100 cycles (Figure 4e). In addition, DA‐NCM cathode exhibits stable energy density retention (Figure S31) and a desirable fast charging (5 C) performance (Figure S32) at 4.5 V.

The loss of reversible capacity of NCM cathodes occurs mainly at high voltage due to the deterioration of microstructure and interfacial side reactions. Therefore, the high‐voltage stability of the NCM affects its cyclability. From the dQ/dV curves of different cycles, the H2‐H3 redox peak at high voltage in DA‐NCM retains both intensity and voltage position, indicating stable phase transition behavior. By contrast, the peak in P‐NCM is significantly suppressed, reflecting reduced reversibility (Figure S33) [47]. Meanwhile, compared with P‐NCM, DA‐NCM maintains highly overlapping voltage‐capacity curves during long‐term cycling (Figure S34) and exhibits lower voltage polarization (Figure S35). As a result, DA‐NCM delivers remarkable cycling stability and retains its structural integrity during cycling.

### 2.3 Controlled Lattice Breathing Achieved Through Heterogeneous Coatings

The lattice mismatch between the heterogeneous coating and NCM would suppress anisotropic lattice expansion and contraction while also reduce mass transport kinetics, leading to capacity decay [19]. Therefore, the trade‐off effects among samples with different ratios of dual‐architecture modifications must be considered. According to the comparison results of lithium‐ion diffusion coefficients in the galvanostatic intermittent titration technique (GITT) test (Figures 4c and S36a), all samples exhibit uneven kinetic reduction during high‐voltage charge/discharge, with a sharp decline occurring during deep de‐lithiation above 4.1 V, specifically during the H2‐H3 phase transition. Among these samples, DA‐NCM demonstrates the highest apparent lithium‐ion diffusion coefficient during the entire voltage range. The dQ/dV curves derived from GITT (Figures 4f and S36b) confirm that introducing heterogeneous coatings at different ratios indeed creates nondiffusive energy barriers, which may lead to capacity decline. However, the intrinsic thermodynamic behavior of DA‐NCM is most similar to that of P‐NCM, indicating that its moderate coating layer does not excessively constrain the cathode material's lattice breathing. Thermodynamic equilibrium diffusion diagrams for different samples also confirm this mild phase transition regulation effect of DA‐NCM (Figure S37).

Further in‐situ XRD testing reveals that both P‐NCM and DA‐NCM exhibit distinct continuous hexagonal phase transitions during charging and discharging (Figures 4g and S38) [48]. When the charging voltage exceeds 4.0 V, the (003) diffraction peak of P‐NCM significantly deviates from its original 2θ position, indicating a sharp contraction of the c‐axis, which leads to the accumulation of irreversible structural deformation and associated stresses during repeated delithiation/lithiation cycles (Figure 4h). By contrast, the variation in the c‐axis is reduced in DA‐NCM (Figure 4i), indicating that the mechanical constraint effect of the heterogeneous coating can indeed effectively mitigate the anisotropic lattice expansion and contraction during the charge and discharge process. Surprisingly, unlike other studies, the (003) peak of DA‐NCM exhibits broadening during charging (Figure S39). This broadening is usually considered as a harmful two‐phase coexistence caused by hindered lithium‐ion diffusion and would exacerbate the irreversibility of the H2‐H3 phase transition [49]. However, our findings confirm that the dual‐architecture coating design can transform the originally abrupt and severe phase transition into a mild, controllable phase transition, spreading it across a wider voltage window and thereby facilitating stress adaptation. Therefore, the (003) diffraction peak of DA‐NCM returns to its original position after the cycle, achieving high phase reversibility (Figure 4i). Combined with the apparent lithium‐ion diffusion coefficient and GITT‐dQ/dV results, it is confirmed that a moderate coating on DA‐NCM affords efficient surface ion transport kinetics and moderates lattice breathing to a mild, controllable regime. This approach achieves optimal balance between kinetic performance and structural stability by constraining anisotropic lattice expansion and contraction without capacity loss. In contrast, P‐NCM exhibits strong lattice breathing, generating significant anisotropic expansion/contraction and enormous internal stresses. Its (003) diffraction peak fails to recover the initial position after complete discharge, as shown in Figure 4h. This irreversible peak shift indicates that stresses induced by the lattice breathing are released through permanent structural damage, potentially manifesting as lattice slip, dislocation growth, or microcrack formation [46].

To quantify lattice breathing and the H2‐H3 phase transition behavior, we performed further processing on the in situ XRD data. Figure 4j,k show the (003) diffraction patterns and their corresponding deconvoluted plots in the voltage range from 4.0 to 4.5 V. Upon charging beyond 4.0 V, the lattice contracts with delithiation, and the (003) diffraction peak shifts to higher angles. At 4.2 V, both P‐NCM and DA‐NCM retain an inactive residual H2 phase. For P‐NCM, this is due to surface passivation, whereas for DA‐NCM, it is due to the mechanical anchoring of the coating. Toward the end of charging, H2 and H3 coexist in both the P‐NCM and DA‐NCM. Although the area of the H3 phase peak is larger in DA‐NCM, the extent of c‐axis contraction induced by the H2‐H3 phase transition (Δθ’, defined as the angular displacement between H3 (4.5 V) and H2 (4.0 V) after deconvolution) is smaller in DA‐NCM than that in P‐NCM throughout the charging process, indicating that the milder H2‐H3 phase transition of DA‐NCM. The quantitative peak analysis data from in situ XRD are shown in Table S15. The c‐axis contraction during the phase transition is a key parameter for generating electrochemically induced defects and causing structural degradation [50]. Combining the fact that the lattice contraction of DA‐NCM during full charging (Δθ, defined as the angular displacement between H2 (4.5 V) and H2 (4.0 V) after deconvolution) is also minimal, with a slight increase in the residual inactive H2 phase at the end of charging, the following conclusion can be drawn: Due to the mechanical pinning effect, DA‐NCM exhibits less lattice contraction during charging caused by delithiation and the H2‐H3 phase transition at the end of charging, thereby achieving mild and controllable lattice breathing.

To evaluate the balance between the kinetic degradation caused by the heterogeneous coating and the inherent structural degradation on the NCM surface, we conducted in situ XRD testing on P‐NCM and DA‐NCM exposed to air for 30 days. The results reveal that P‐NCM exhibits residual inactive H2 phase during charging, while the (003) peak of DA‐NCM smoothly shifts to higher angles (Figure S40). Therefore, we conclude that the non‐diffusive energy barrier introduced by the heterogeneous coating is acceptable, as it delivers greater gain effects during long‐term cycling.

### 2.4 Surface and Bulk Structural Stability of Dual‐Architecture Designs

Further investigation of post‐cycling structural stability is crucial for understanding the structural evolution of electrode materials during electrochemical cycling. As depicted in Figure S41a,b, a thick and inhomogeneous CEI is formed, and a structural rearrangement layer of 10‐30 nm appears on the surface of P‐NCM, which is attributed to the spinel structure and the rock‐salt structure. However, DA‐NCM still maintains a homogeneous layered structure. A thin LLNO phase is still tightly wrapped around the surface while forming a thin and uniform CEI layer of only 2.91 nm, indicating that the designed anchored oxygen‐release‐suppression phase improves the surface structure stability of the DA‐NCM (Figure S41c, d). The degree of secondary particle fracture in DA‐NCM after high‐rate cycling is lower, as shown in Figures 5a,b and S42. In addition, XRD results show that the peaks of DA‐NCM are more intense and sharper, and the I_(003)_/ I_(104)_ ratio is higher than that of P‐NCM, indicating a stable structure and a lower degree of Li/Ni cation disorder (Figures S43 and 5c). Moreover, the characteristic peaks of LLNO could still be observed in DA‐NCM, suggesting that LLNO is stably anchored on the DA‐NCM surface (Figure 5d). For the unanchored La‐NCM, the LLNO coating has already peeled off during long‐term high‐rate cycling, demonstrating the effectiveness of the LaMO anchoring strategy (Figure S44 and Table S16). In addition, in situ electrochemical impedance spectroscopy (EIS) results indicate that surface structure degradation (R‐3m to Fd‐3m to Fm‐3m) in deep charge state [6, 51] of DA‐NCM is mitigated under deep charge states, with stable R_ct_ during charging. By contrast, P‐NCM exhibits a significantly increased R_ct_, especially in a deep charge state (Figure S45 and Table S17). Thus, the optimization of surface structure and microscopic morphology brought by the anchored strategy could effectively improve structural reversibility for DA‐NCM cathodes.

**FIGURE 5:**
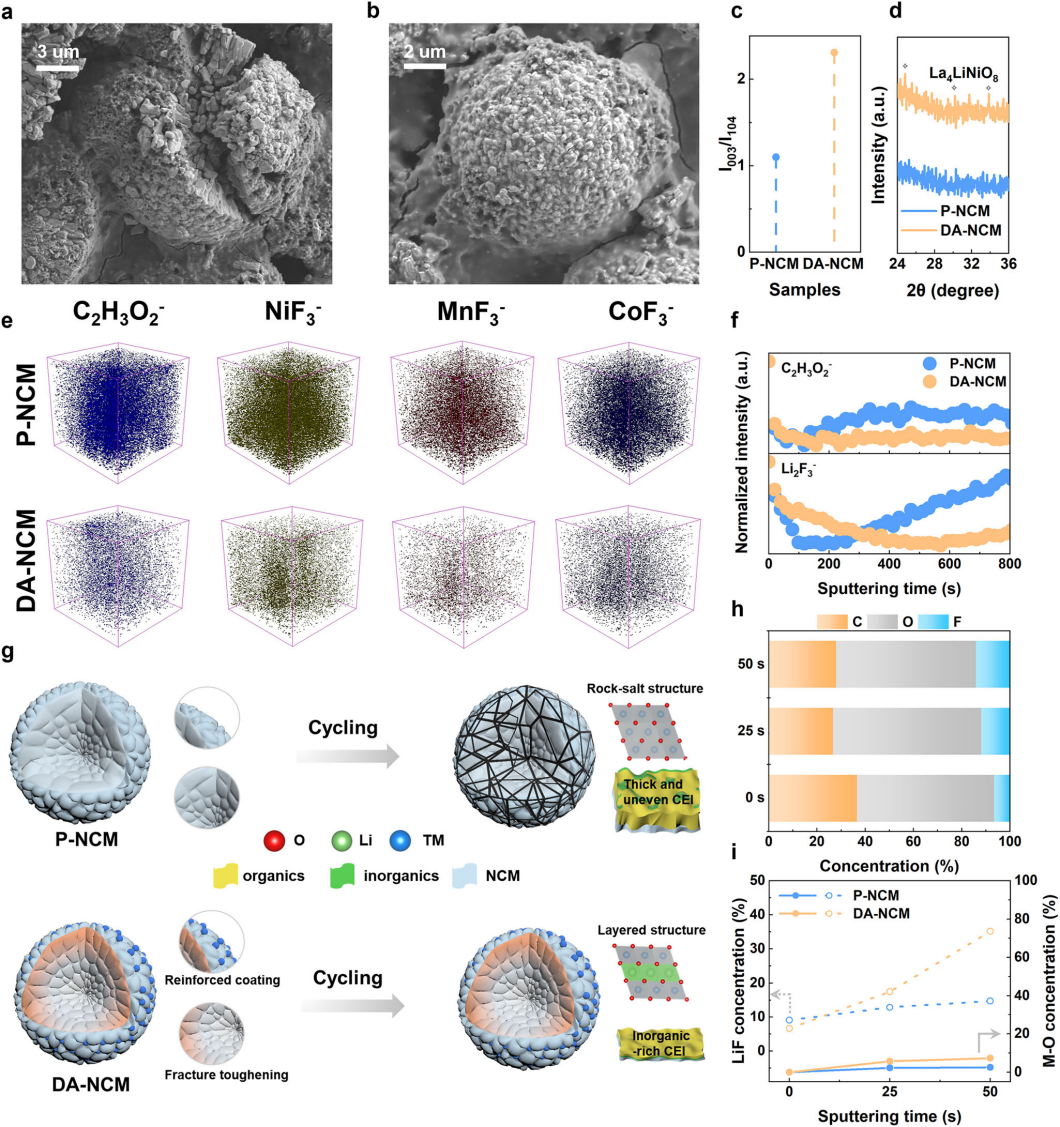
Enhanced surface and mechanical structural stability due to an anchored oxygen‐release‐suppression phase strategy. SEM images of P‐NCM (a) and DA‐NCM (b) after high‐rate cycling. (c) I_003_/I_104_ of P‐NCM and DA‐NCM after high‐rate cycling. (d) Magnified XRD patterns of P‐NCM and DA‐NCM after high‐rate cycling. (e) 3D rendering TOF‐SIMS fragments of P‐NCM (top) and DA‐NCM (bottom). (f) TOF‐SIMS depth profiles of C_2_H_3_O_2_^−^ and Li_2_F_3_^−^ for P‐NCM and DA‐NCM after cycling. (g) Schematic diagram of anchored strategy to enhance the interfacial structural stability and mechanical stability. (h) C, O and F atomic concentrations of DA‐NCM after cycling. (i) Comparison of LiF and M‐O concentration changes during the etching process.

Time of flight secondary ion mass spectrometry (TOF‐SIMS) testing was performed to verify the suppression of interfacial side reactions on the cathode surface. As shown in Figure 5e, DA‐NCM exhibits lower contents of inorganic fragments NiF_3_^−^, CoF_3_^−^, and MnF_3_^−^ generated by transition metal dissolution. Additionally, it exhibits lower levels of organic C_2_H_3_O_2_^−^ fragments generated from surface electrolyte decomposition, which also decline more rapidly to a stable state. This confirms the more stable surface and thinner CEI of DA‐NCM. The LiF components represented by Li_2_F_3_^−^ enrich within the DA‐NCM CEI layer without penetrating the cathode interior (Figure 5f). The combined analysis of the interface and surface confirms that our designed coating enhances the interface and surface stability of high‐nickel cathodes.

The results of Ar‐etched XPS also support the same conclusion. Before etching, the surface of P‐NCM exhibits more organic species (e.g., ─CH_2_─, C─O), originating from electrolyte decomposition, whereas such components are less abundant on DA‐NCM (Figure S46). DA‐NCM surface before etching exhibits lower concentrations of Li_x_PO_y_F_z_ and LiF generated from interfacial side reactions, indicating the enhanced interfacial stability of DA‐NCM [52]. As shown in Figure 5h and Figure S47, both DA‐NCM and P‐NCM exhibit increased F content and reduced C content after etching, indicating a CEI layer composed of an outer layer rich in organics and an inner layer rich in inorganics. In terms of specific components, the C concentration of DA‐NCM decreased from 36.6% to 27.9% (37.6% to 34.9%, P‐NCM), which indicates fewer organic components in its CEI. Furthermore, as shown in Figure 5i, DA‐NCM exhibits a progressively higher concentration of inorganic LiF with increasing etching depth, consistently surpassing that of P‐NCM. This indicates a more robust inorganic CEI layer, which is advantageous due to LiF's low ionic transport barriers and superior strain tolerance [53]. The higher metal‐O (M─O) concentration in DA‐NCM suggests a thinner CEI layer on its surface [54]. This thin inorganic‐rich CEI would promote ion migration and prevent DA‐NCM from being eroded by organic solvents. The EIS test of cycled DA‐NCM exhibits lower R_ct_ and CEI impedance (R_CEI_), which also supports the same conclusion (Figures S48 and S49). Such a series of in‐depth studies of the cycled cathodes confirms that our dual architecture anchoring strategy could derive high‐quality CEI and suppress irreversible surface degradation thus effectively improving the mechanical and structural stability of DA‐NCM (Figure 5g).

### 2.5 Analysis of DA‐NCM for Regulating Oxygen Activity and Anchoring Mechanisms

To evaluate oxygen suppression performance, all samples were subjected to cycling tests under high‐voltage and elevated‐temperature conditions. As shown in Figures 6a and S50, DA‐NCM exhibits an outstanding electrochemical performance with a capacity retention of 91.5% after 200 cycles under 2.8‐4.3 V at 1 C, 50°C, which is far higher than that of P‐NCM (56.9%) and La‐NCM (67.9%). Furthermore, DA‐NCM maintains satisfactory electrochemical stability even when the voltage is increased to 4.5 V, sustaining 71.6% capacity retention after 150 cycles (Figure S51). EPR analysis of thermally damaged cathode powders before cycling shows that P‐NCM contains more oxygen vacancies than DA‐NCM, further confirming that the dual‐architecture coating enhances lattice oxygen stability rather than merely serving as a passive surface barrier (Figure 6b). Differential electrochemical mass spectrometry (DEMS) was employed to in‐situ monitor gas evolution during charging and discharging. As shown in Figure 6c, P‐NCM starts to release CO_2_ and O_2_ at 3.972 V, and CO is produced simultaneously when the voltage increases (using the galvanostatic charging model). Subsequently, potentiostatic charging under high‐voltage conditions is applied, during which gas emissions rose sharply. The dominant gas detected is CO_2_, indicating that severe electrolyte decomposition occurred during this process. This observation aligns with the fact that EC solvents are prone to decomposition at high voltages. Significant oxygen evolution is also observed during this process, which is attributed to the evolution of lattice oxygen. In contrast, Figure 6d displays that the gas emission of the DA‐NCM is significantly decreased and delayed to 4.015 V. The cumulative oxygen release of DA‐NCM was reduced to half of that of P‐NCM (Figure S52). These comparative results clearly demonstrate the excellent oxygen release suppression performance provided by DA‐NCM. Semi‐quantitative XPS analysis shows that the oxygen vacancy content in the LLNO and LaMO coatings on the DA‐NCM surface were 12.68% and 18.14%, respectively (Figure S53). When scaled to the entire material, the upper limit of oxygen vacancy capture in the coatings ranged from 0.0158% to 0.0454% (molar ratio). Furthermore, based on DEMS testing, the oxygen vacancy concentration in the coating was approximately 6.5‐9.31 µmol g^−1^, with an oxygen storage capacity of 3.25‐4.66 µmol g^−1^, which is lower than the cumulative gas evolution of approximately 20 µmol g^−1^ during the initial charging process of DA‐NCM. Therefore, oxygen vacancies in the coating mainly provide oxygen‐buffering capacity in the first cycle. In contrast, the sustained suppression of oxygen release during cycling is primarily ascribed to the improved stability of lattice oxygen in DA‐NCM.

**FIGURE 6:**
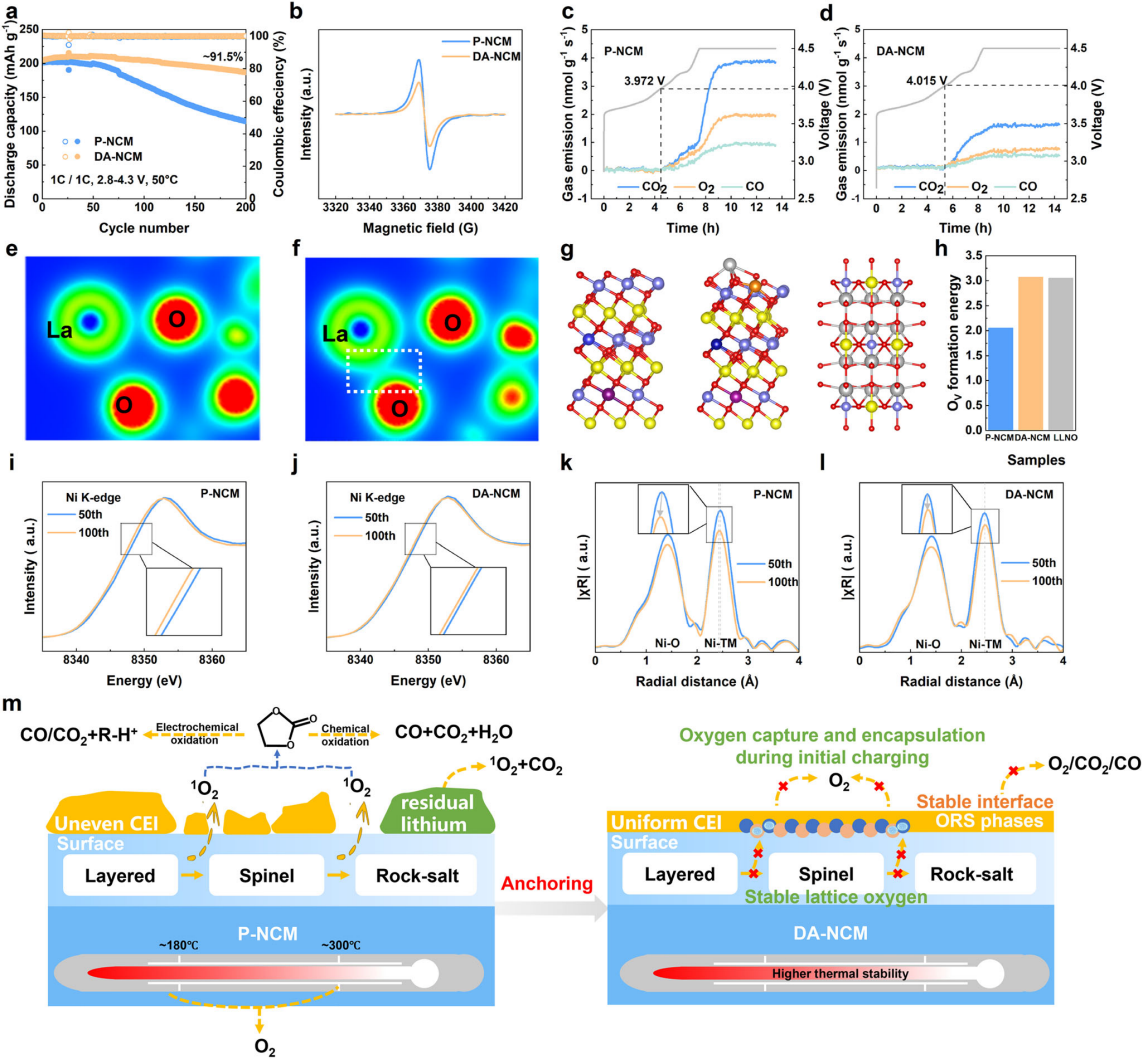
Stable coating and suppressed oxygen production. (a) Cycling performances of P‐NCM and DA‐NCM under 2.8‐4.3 V at 50°C. (b) EPR results after thermal destruction of the cathode. (c, d) In situ DEMS profiles of P‐NCM (c) and DA‐NCM (d). (e, f) 2D slices of the charge density distribution of unanchored La─O structure (e) and anchored La─O structure (f). (g, h) Structural diagrams of P‐NCM (left), DA‐NCM (middle), and LLNO (right) for DFT calculations (g), along with their corresponding oxygen vacancy formation energies (h). (i, j) Ni K‐edge XANES spectra for 50th and 100th cycled electrodes of P‐NCM (i) and DA‐NCM (j). (k, l) EXAFS spectra for 50th and 100th cycled electrodes of P‐NCM (k) and DA‐NCM (l). (m) Diagram of gas production inhibition by anchored strategy (^1^O_2,_ singlet oxygen; ORS phase, oxygen‐release‐suppression phase).

DSC tests were further employed to verify the intrinsic thermal stability of DA‐NCM and to confirm its lattice oxygen stability (Figure S54). DSC test results for fresh and cycled cathode powders show that DA‐NCM exhibits a higher onset exothermic temperature and lower heat output than P‐NCM both before and after cycling, reflecting enhanced thermal robustness. This also indicates that the phase transitions and oxygen evolution reactions occurring in DA‐NCM at high temperatures are reduced [55]. To further correlate thermal stability with gas evolution behavior, pouch cells based on both materials were assembled and tested during the initial charging process at 50°C. The pouch cell assembled with the P‐NCM cathode exhibits significant swelling, in stark contrast to the DA‐NCM cell, which shows no detectable gas release (Figure S55). In Raman spectroscopy, the E_g_ and A_1g_ vibrational mode peaks of DA‐NCM shift toward higher wavenumbers, potentially attributable to enhanced TM‐O bonding [56] and stress transfer from the tightly anchored surface layer [57], corresponding to DA‐NCM's higher thermal runaway temperature (Figure S56). These findings indicate that the anchored oxygen‐release‐suppression phase plays a critical role in enhancing the safety of Ni‐rich cathodes. Consequently, DA‐NCM shows reduced oxygen release, broader application potential, and outstanding electrochemical performance (Figure S57).

DFT calculation was carried out to elucidate the anchoring mechanism and the regulation of oxygen activity. The bonding effect between the La atom and surface oxygen was analyzed. Since the cations within the coating are usually coordinated to the surface oxygen, we predict a feasible way of bonding between La, O, and Mo atoms (Figures S58 and 6g, middle). By calculating the energy states of O atoms directly coordinated to La, it is observed that, compared to the unanchored La─O in La‐NCM, the oxygen in DA‐NCM exhibits fewer high‐energy states near the Fermi level (E_F_), indicating a stabilization of the O electronic structure, which may contribute to improved interfacial and thermal stability (Figure S59). Owing to the higher bond strength compared to Ni─O, the La─O interactions facilitate charge transfer from La to lattice oxygen, effectively stabilizing the oxygen and mitigating its participation in parasitic reactions (Figure S60). As shown in the 2D differential charge density slices (Figure S61), compared to the La‐NCM sample, the DA‐NCM sample exhibits a noticeable aggregation of electron clouds in the La─O bonding region, confirming the enhanced La─O bonding [30]. Furthermore, the charge density plot reveals stronger electron cloud overlap in the anchored La─O bond of DA‐NCM, with the oxygen electron cloud polarized toward La, indicative of a covalent bonding character (Figure 6e,f) [5]. Such a stronger covalent La─O bond would further increase the electronegativity and stability of oxygen and simultaneously achieve the anchoring of the LLNO phase.

To further elucidate the mechanisms of oxygen stabilization and trapping, oxygen vacancy formation energies were calculated as shown in Figure 6g,h. Comparison of the NCM (001) surface before and after modification shows that the modified NCM exhibits a higher oxygen vacancy formation energy, indicating that oxygen release is thermodynamically less favorable and that lattice oxygen stability is enhanced. The oxygen vacancy formation energy of the primary LLNO coating was also calculated and found to be higher than that of pristine NCM, suggesting that lattice oxygen release from LLNO requires greater energy input. In other words, during the process by which oxygen atoms migrate from the NCM lattice to the LLNO lattice and are subsequently released into the air as gas, the energy transfer between NCM and LLNO (E (transfer) = E (NCM) – E (LLNO)) = ‐1.018 eV < 0. Therefore, after oxygen is released from the NCM lattice during cycling, its incorporation into the LLNO coating represents an active oxygen capture process rather than simple physical blocking.

The overall Ni valence and coordination environment were investigated using the XANES technique with high‐energy hard x‐rays. After 50 and 100 cycles, the Ni K‐edge of the fully charged P‐NCM shifted to a lower energy position compared with the DA‐NCM. This behavior is attributed to a reduction in the valence state of Ni ions, which occurs as a result of oxygen release from the cathode structure (Figure 6i,j) [58]. Extended x‐ray absorption fine structure (EXAFS) data in Figure 6k,l show that the Ni‐TM coordination distance of P‐NCM is shortened after cycling, indicating lattice shrinkage and defect creation [59]. In contrast, the Ni‐TM coordination distance in DA‐NCM remains unchanged. In addition, the decrease of the Ni‐TM peak of cycled DA‐NCM is also significantly smaller than that of P‐NCM, proving its highly stable local coordination environment. In the wavelet‐transformed EXAFS analysis, the Ni─O bond in P‐NCM exhibits a shift toward higher *k* values, whereas the Ni─O bond position in DA‐NCM remains unchanged, suggesting the reduction of oxygen‐related defects in DA‐NCM (Figure S62) [17]. Therefore, the remarkable cycling stability of DA‐NCM under high voltage and high temperature would be attributed to the following reasons. Firstly, the LLNO phase could be firmly anchored to the NCM surface by the LaMO phase without detaching. Meanwhile, charge transfer from the La ion would improve the oxygen electronegativity to resist over‐oxidation during high‐voltage charging thus improve the thermal stability of lattice oxygen. Secondly, the oxygen‐vacancy‐rich LaMO and LLNO phases with high oxygen vacancy formation energy would build an oxygen barrier layer on the surface of DA‐NCM, realizing the capture and encapsulation of reactive oxygen during the initial charging process [9, 17]. Thirdly, this anchored strategy could suppress the bulk, surface and interfacial structure deterioration in the deep charge state, thereby reducing the release of reactive oxygen species and the subsequent gas‐producing reactions [60]. The mechanism is illustrated in Figure 6m. Notably, compared to DA‐NCM, the worse high‐temperature and high‐voltage performances of the samples with La and Mo elements introduced sequentially in two steps or single LaMO coated NCM further demonstrate the effectiveness of this one‐step anchoring strategy (Figure S63). Furthermore, this dual‐architecture strategy is scalable and can be applied to Ni‐rich cathode materials with higher nickel content (LiNi_0.95_Co_0.04_Al_0.01_O_2_, NCA95). As shown in Figure S64, DA‐NCA prepared using an optimized sintering mechanism still forms LaMO and LLNO and exhibits excellent high‐voltage stability (Figure S65). The advantages of the one‐step method for synthesizing dual‐architecture materials are evident in several aspects. First, the one‐step method simplifies the production process and enables the synthesis of a wide range of materials. Furthermore, the one‐step method is crucial for the synthesis of LaMO as the anchoring phase, as it enables the simultaneous formation of pure LaMO rich in complete oxygen vacancies, thereby constructing a unique and intact NCM‐LaMO‐LLNO interface and forming stable La─O bonds, without compromising the lattice order of the bulk NCM. It is difficult to achieve both of these goals at the same time using the two‐step method (Figures S66–S73). In addition, the one‐step‐derived dual architecture exhibits enhanced thermal stability (Figure S54a), further confirming the strengthened metal‐oxygen bonding within the structure. This dual‐phase strategy can also be extended to Ni‐rich cathodes with higher Ni contents.

## 3 Conclusions

In summary, we report a dual‐architecture design strategy for Ni‐rich NCM (DA‐NCM), achieved through a one‐step synthesis. An oxygen‐release‐suppression phase (La_4_LiNiO_8_, LLNO) is anchored on the bulk surface and stabilized by the pinning effect of a secondary inert phase (La_2_Mo_2_O_9_, LaMO), forming an oxygen vacancy‐rich interface. Theoretical calculations and in situ characterizations confirm that the LaMO phase anchors the LLNO coating through a strengthened La─O bond pinning effect, preventing the detachment of LLNO. This design effectively suppressed the release of oxygen by predominantly enhancing the thermodynamic stability of lattice oxygen, with only a minor, transient contribution from the capture of released oxygen species at surface vacancies during the initial charging step. Moreover, the introduction of the anchored oxygen‐release‐suppression phase significantly regulates the mild phase transition of DA‐NCM and suppresses surface side reactions, thereby enhancing the bulk/interfacial structural stability of DA‐NCM. DA‐NCM cathodes exhibit a high‐capacity retention of 95.7% after 200 cycles at 4.3 V, and 93.6% capacity retention after 200 cycles when the operating voltage range is extended to 4.5 V. The capacity retention of 91.5% has been achieved after 200 cycles even at 50°C. The oxygen release is reduced by half, which can significantly extend the cycle life of the batteries. This work integrates multiple strategies to stabilize Ni‐rich NCM cathodes and reveals the synergistic effects among them, thereby offering valuable insights into the development of next‐generation high‐energy cathode materials with enhanced longevity and safety.

## Author Contributions

**Ziqi Liu**: writing – review and editing, writing – original draft, conceptualization, software, formal analysis. **Yiming Zhang**: writing – original draft, validation. **Yong Chen**: writing – review and editing, conceptualization, formal analysis, software. **Meng Yao**: writing – review and editing, writing – original draft, funding acquisition, conceptualization, methodology. **Yangyang Wang**: data curation. **Du Yuan**: validation, formal analysis. **Haitao Zhang**: project administration. **Yun Zhang**: writing – review and editing, funding acquisition, investigation, supervision, resources. **Guoxiu Wang**: resources, supervision, project administration, writing – review and editing, investigation.

## Conflicts of Interest

The authors declare no conflicts of interest.

## Supporting information




**Supporting File**: anie74211‐sup‐0001‐SuppMat.docx.

## Data Availability

The data that support the findings of this study are available from the corresponding author upon reasonable request.
