# Supplementary material for: Anchored Oxygen‐Release‐Suppression Phase Stabilizing High‐Voltage Ni‐Rich Cathode Materials
Source: Angew Chem Int Ed Engl. 2026 Aug 23;65(42):e9916183. doi: 10.1002/anie.9916183 (PMC13653588; doi:10.1002/anie.9916183)
Supplement: Supplementary file 1 [file ANIE-65-e9916183-s001.docx]

Supplementary Information

Anchored Oxygen-Release-Suppression Phase Stabilizing High-Voltage Ni-Rich Cathode Materials

Ziqi Liu^1,5^, Yiming Zhang^1,5^, Yong Chen^2,5^, Meng Yao^1*^, Yangyang Wang^1^, Du Yuan^3^, Haitao Zhang^4^, Yun Zhang^1*^, and Guoxiu Wang^2*^

1 College of Materials Science and Engineering, Sichuan University 610064 Chengdu (P. R. China).

E-mail: yaomeng@scu.edu.cn; [y_zhang@scu.edu.cn](mailto:y_zhang@scu.edu.cn).

2 Centre for Clean Energy Technology, University of Technology Sydney, Broadway, Sydney, NSW 2007, Australia.

E-mail: Guoxiu.Wang@uts.edu.au.

3 College of Materials Science and Engineering, Changsha University of Science and Technology, Changsha 410004, Hunan, China.

4 Institute of Process Engineering, Chinese Academy of Science, Beijing 100086, Beijing, China.

5 These authors contributed equally to this work.

Keywords: Ni-rich cathode, anchored strategy, dual architecture, oxygen emission, structural stability.

Methods

Materials synthesis

Pristine LiNi_0.9_Co_0.05_Mn_0.05_O_2_ (P-NCM) was obtained by mixing Ni_0.9_Co_0.05_Mn_0.05_(OH)_2_ precursor (SHENZHEN KEJING) and LiOH·H_2_O (CHRON CHEMICALS) compound with a 1:1.05 molar ratio, then calcined at 500 °C for 6 h and 740 °C for 12 h (5 °C min^-1^) in an oxygen atmosphere. For the preparation of modified NCM, labeled LM03-NCM (0.3 mol% La and 0.3 mol% Mo), DA-NCM (0.5 mol% La and 0.5 mol% Mo), LM10-NCM (1 mol% La and 1 mol% Mo), La-NCM (0.5 mol% La) and Mo-NCM (0.5 mol% Mo) respectively, appropriate amounts of La(NO_3_)_3_·6H_2_O (aladdin) and (NH_4_)_6_Mo_7_O_24_·4H_2_O (JSHAN CHEMICAL TEST) were each dispersed/dissolved in anhydrous ethanol under stirring with continuous stirring and ultrasonic dispersion. The ethanol solutions of Mo and La were sequentially dropped into the anhydrous ethanol dispersion of the precursors with slow stirring and then evaporated to dryness at 90 °C in a water bath. The resulting powder was blown dry at 60 °C for 12 h and then mixed with a lithium source for sintering, which was the same process as P-NCM. For the double-step modified NCM (DS-NCM), the precursor and Mo source were first mixed in anhydrous ethanol, dried completely, and then sintered with LiOH·H_2_O at 500 °C for 6 h and 740 °C for 12 h (5 °C min^-1^) in an oxygen atmosphere. The obtained product was then mixed with the La source in ethanol and sintered in oxygen for 5 h at 500 °C or for 10 h at 700 °C. All the DS-NCM samples in the figures that do not have the temperature indicated are samples subjected to 500 °C heat treatment. For pure LaMO-coated NCM sample (LaMO-NCM), after synthesizing NCM, it was mixed with La and Mo sources and sintered at 500 °C for 12 hours. For pristine LiNi_0.95_Co_0.04_Al_0.01_O_2_ (P-NCA) and NCA modified using a dual-architecture strategy (DA-NCA), the Ni_0.9_Co_0.05_Mn_0.05_(OH)_2_ precursor (SHENZHEN KEJING) was used for preparation and the remaining process steps are the same as those for P-NCM and DA-NCM. The optimized sintering mechanism employed was 500 °C for 6 h and 740 °C for 12 h (5 °C min^-1^) in an oxygen atmosphere.

Physical characterizations

Crystal structures of the as-synthesized samples were detected by X-ray diffraction (XRD, DX-2700BH) at a scan rate of 9 ° min^−1^ and 0.6 ° min^−1^ and by neutron powder diffraction (NPD, China Spall ation Neutron Source). The Rietveld refinement of the slow-scan XRD data and NPD data were performed by the GSAS Ⅱ program. The in-situ XRD measurements during charging-discharging of as-prepared samples were carried out using a DteX250 (H) X-ray diffractometer equipped with Cu Kα radiation. The diffraction patterns were collected in the scattering 2θ angle range of 10-80°. Focused ion beam (FIB, Thermo Scientific Helios 5 CX), scanning electron microscope (SEM, Thermo Scientific Scios 2C), energy dispersive X-ray spectrometer (EDS, OXFORD ULTIM Max65), high-resolution transmission electron microscope (HRTEM, JEOL JEM-F200) combined with a high-angle annular dark field scanning transmission electron microscopy (HAADF-STEM, Thermo Scientific spectra 300 S) and electron energy loss spectroscopy (EELS, Gatan 1065) to analyze the particle morphology, microstructure and element distribution of the materials. X-ray photoelectron spectroscopy (XPS, Thermo Fischer, ESCALAB 250Xi), hard X-ray absorption spectroscopy (XAFCA beamline of Singapore Synchrotron Light Sources), soft X-ray absorption spectroscopy (Taiwan Light Source of National Synchrotron Radiation Research Center), and time of flight secondary ion mass spectrometry (TOF-SIMS, PHl nanoTOE II) were applied to analyze the distribution of elements and valence states of cathode materials. Nitrogen isothermal adsorption-desorption testing (Micromeritics ASAP 2460) is used to measure the specific surface area of different cathodes. Electron paramagnetic resonance (EPR, EMX-PLUS, BrookeA300) was used to characterize the oxygen vacancies in the cathode materials. To determine the thermal stability of the cathode material, differential scanning calorimetry (DSC, DSC Q2000) and Raman spectra (Horiba LabRAM HR Evolution) were performed on the cathode. The in-situ differential electrochemical mass spectrometry (DEMS, QMG220) was applied to detect and characterize the gas emission of the prepared samples during the first charging process.

Electrochemical measurements

Cathodes were prepared by mixing 80 wt.% active material, 10 wt.% conductive carbon, and 10 wt.% PVDF dissolved into N-Methyl-2-pyrrolidinone (NMP). Afterward, the slurry was coated onto aluminum foil and dried at 120 °C (12 h) in a vacuum box. The cathode loading level was ~3 mg cm^-2^ and 20 mg cm^-2^. CR2032 half-cells were assembled with 12 mm diameter cathode tablet, 1M LiPF_6_ dissolved in ethylene carbonate (EC), ethyl methyl carbonate (EMC), and dimethyl carbonate (DMC) with a volume ratio of 1:1:1 as the electrolyte, Celgard 2500 separator, and Li foils in an argon-filled glove box (KH1200), The half-cells were tested in the voltage range of 2.8-4.3 V/2.8-4.5 V at different current density (1 C = 200 mA g^-1^) at different temperatures. The EIS test was assessed with a voltage amplitude of 5 mV and a frequency range of 100 kHz to 0.01 Hz. The galvanostatic intermittent titration technique (GITT) was used to characterize the Li^+^ diffusion behavior of the cathode material at the voltage of 2.8-4.3 V and 2.8-4.0 V with a titration step at 0.1 C of 10 min and a relaxation step of 30 min. The thermodynamic equilibrium potential (relaxation OCV) was tested to eliminate interference from active material mass and verify consistency between actual SOC and measured capacity. 30 cycles were conducted at 0.1 C within the 2.8-4.5 V voltage range, with an immediate 1-hour open-circuit voltage (OCV) step added after each charge to 4.5 V, repeated every five cycles. Cyclic voltammetry (CV) test was performed at a sweep speed of 0.1 mV s^-1^ at a voltage of 2.8-4.3 V on a CHI660D electrochemical workstation. For the preparation of the pouch cell, the DA-NCM cathode, Li anode, and Celgard 2500 separators were assembled in the argon-filled glove box, the cell was sealed after filling with electrolyte. 1 M LiPF_6_ dissolved in a 1:1:1 (v/v) EC/DMC/EMC mixture was used as the electrolyte. The cathode loading level was ~4 mg cm^-2^. The pouch cells were tested in the voltage range of 2.8-4.3 V at 0.5 C. For energy density calculation, the total mass includes the mass of cathode, anode, and electrolyte, is 40.449mg. Pouch cell capacity is ~4.821mAh, and the discharge voltage is ~ 3.817V. For coin NCM/graphite full cells, the cathode preparation method remains unchanged. The anode is prepared by mixing 80 wt.% active material, 10 wt.% conductive carbon, and 10 wt.% PVDF dissolved in NMP. The slurry was then coated onto copper foil and dried for 12 hours at 120 °C in a vacuum box. The specific loading of the cathode used in the full cell was ~10 mg cm⁻², and the negative to positive capacity ratio (N/P ratio) was controlled at 1.10 ± 0.01. Full-cell testing was conducted at room temperature within a voltage range of 2.8–4.2 V or 2.8–4.5 V.

Theoretical methods

The density functional theory (DFT) calculation was performed using the Vienna ab initio Simulation Package (VASP) with the projector augmented-wave potentials^1^. The generalized gradient approximation (GGA) with the Perdew-Burke-Ernzerhof (PBE) functional^2^ was employed to describe the exchange-correlation. A cutoff energy for the plane-wave basis was set to 550 eV for all calculations. The energy convergence criteria for self-consistent-field iteration were 10^-4^ eV, and the atomic positions were fully optimized until all the residual forces were smaller than 0.01 eV Å^-1^.

Figure S1. XRD pattern (a), TEM image (b) and corresponding SAED pattern (c) of LaMO obtained using optimized La/Mo ratio and sintering process.

Figure S2. Raman spectroscopy results of LMO obtained using optimized La/Mo ratio and sintering process.

Figure S3. EPR results of LaMO coated NCM (LMO-NCM) and P-NCM.

Figure S4. XRD patterns of P-NCM, La-NCM and DA-NCM.

Figure S5. XRD Rietveld refinement for P-NCM (a), La-NCM (b) and DA-NCM (c).

Figure S6. NPD Rietveld refinement for P-NCM (a), La-NCM (b).

Figure S7 XRD patterns for P-NCM and different ratios modified samples at a fast scan rate of 9° min^−1^.

The zoom peaks on the right figures show that the 003 peak shifts to a lower angle and there are obvious LLNO diffraction peaks after the anchored strategy. Besides, this anchored strategy reduces Li/Ni mixing compared with P-NCM, La-NCM and Mo-NCM.

Figure S8 XANES spectra of Ni K-edges of P-NCM and DA-NCM.

Figure S9. XPS data of Ni 2p (a), O 1s (b), Mo 3d (c), La 3d (d), Co 2p (e) and Mn 2p (f) taken from the surface of all the samples.

Figure S10. XPS depth profiles Mo 3d of DA-NCM as a function of Ar etching time from 0 s to 150 s.

Figure S11. I_S_/I_M_ ratio of La-NCM and DA-NCM.

Figure S12. EPR results of P-NCM, La-NCM and DA-NCM.

Figure S13. Distribution of Ni obtained from cross-sectional line scanning of P-NCM and DA-NCM.

Figure S14. SEM images of P-NCM (a) and DA-NCM (b).

Figure S15. EDS mapping images of DA-NCM

Figure S16. SEM and corresponding mapping images for LM03-NCM (a), LM10-NCM (b) and La-NCM (c).

Figure S17. Cross-sectional FIB-SEM images of P-NCM (a, b) and DA-NCM (c, d).

The secondary particle size of DA-NCM is significantly reduced and the primary particle is refined, meanwhile, the intergranular pores are reduced.

Figure S18. Nitrogen adsorption-desorption isotherms curves of P-NCM, La-NCM and DA-NCM.

Figure S19. HRTEM image of the island coating of DA-NCM (a) and corresponding SAED pattern (b).

Figure S20. HRTEM images (a) and corresponding SAED patterns (b) of bulk NCM.

Figure S21. The EDS mapping (a) and IFFT patterns (b) corresponding to Figure 3e.

Figure S22. Element distribution (a) and TEM images (b, c) of La-NCM.

Figure S23. Initial coulombic efficiency and cycling performance of DA-modified (a, c) and simple-modified (b, d) samples.

Figure S24. Test results of the thermodynamic equilibrium potential at 0.1 C.

Figure S25. dQ/dV curves of P-NCM and DA-NCM at the first cycle.

Figure S26. Cycle performance of DA-NCM under 2.8-4.3 V at 1 C.

Figure S27. a. High-voltage cycling performance of high-load half-cells (b) Cycling performance of NCM/graphite at different voltages.

Figure S28. Rate performance of DA-modified (a) and simple-modified (b) samples.

Figure S29. GITT curves and the corresponding lithium-ion diffusion coefficient of P-NCM, La-NCM and DA-NCM at 4.0 V (a, b) and 4.3 V (c, d), the solid circle indicates charging, while the hollow circle indicates discharging.

Figure S30. Nyquist plots for P-NCM, La-NCM and DA-NCM before cycling (a) with corresponding Rct values (b), and lithium-ion diffusion coefficient calculated accordingly (d). (e) Comparison of Nyquist plots for DA-modified samples with different modification ratios before cycling.

Figure S31. Energy density retention of P-NCM and DA-NCM under 2.8-4.5 V at 1 C.

Figure S32. Fast charging performance of P-NCM and DA-NCM under 2.8-4.5 V at 5 C.

Figure S33. dQ/dV curves of DA-modified samples with different modification ratios at extended cycles under 2.8-4.5 V.

Figure S34. Charge/discharge profiles of P-NCM (a) and DA-NCM (b) under 2.8-4.5 V at 1 C.

Figure S35. CV curves of P-NCM (a), La-NCM (b), and DA-NCM (c).

Figure S36. Lithium-ion diffusion coefficient (a) and dQ/dV curves derived from GITT tests of the DA-modified samples with different modification ratios.

Figure S37. Thermodynamic equilibrium phase plots of the DA-modified samples with different modification ratios.

Figure S38. Full range contour plot of in-operando XRD data on P-NCM during the first cycle.

Figure S39. The enlarged (003) peak of P-NCM (a) and DA-NCM (b) during the first charge. Orange and blue represent the charged state at 4.0 V and discharged state at 4.0 V, respectively.

Figure S40. The enlarged (003) peak of air-aged P-NCM (top) and DA-NCM (bottom) during the first charge.

Figure S41. HR-TEM images of the CEI and the structural evolution of P-NCM (a, b) and DA-NCM (c, d) after cycling.

Figure S42. SEM images of P-NCM (a) and DA-NCM (b) after cycling.

Figure S43. XRD patterns of P-NCM and DA-NCM after cycling.

Figure S44. CEI image (a), HR-TEM image of structural evolution (b) and SEM image (c) of La-NCM after cycling. (d) XRD patterns of La-NCM after high-rate cycling (e) The comparison XRD patterns after high rate cycling of P-NCM, La-NCM and DA-NCM.

These cycled-La-NCM tests confirm that although La-NCM could enhance the structural stability to some extent, spinel and rock salt phases still appear on the surface after long cycling, and the CEI is thicker than that of DA-NCM. In addition, cracks could be observed on the particles after high-rate cycling of La-NCM, and the characteristic LLNO peaks are no longer visible after cycling, suggesting that the LLNO coating is detached from the NCM surface after long cycling.

Figure S45. The in-situ galvanostatic electrochemical impedance spectroscopy plots of P-NCM (a) and DA-NCM (b).

Figure S46. Ar-etched XPS data of P-NCM (a-c) and DA-NCM (d-f) after cycling.

Figure S47. C, O and F atomic concentrations of P-NCM after cycling.

Figure S48. Nyquist plots for P-NCM, La-NCM and DA-NCM after cycling (a) and corresponding R_ct_ and R_CEI_ values (b).

Figure S49. Comparison of Nyquist plots for DA-modified samples with different modification ratios after 200 cycles.

Figure S50. Cycling performance of P-NCM, La-NCM and DA-NCM under 2.8–4.3 V at 50 °C.

Figure S51. Cycling performance of P-NCM, La-NCM and DA-NCM under 2.8–4.5 V at 50 °C.

Figure S52. Comparative graph of cumulative gas emissions based on DEMS test results.

Figure S53.O 1s XPS spectra of LLNO (a) and LaMO (b).

Figure S54. DSC test of fresh (a) and cycled (b) cathodes.

Figure S55. Pouch cells of P-NCM (a) and DA-NCM (b) after the initial charge/discharge under 2.8-4.5 V at 50 °C.

Figure S56. Raman spectroscopy of P-NCM, La-NCM and DA-NCM.

Figure S57.Comparison of P-NCM and DA-NCM.

Figure S58. Optimized structure between NCM surface and La and Mo, unanchored La-O structure in La-NCM (unanchored La-O structure).

Figure S59. Density of states for the unanchored (a) and anchored (b) La-O structure.

Figure S60. 3D charge difference of unanchored (a) and anchored (b) La-O structure.

Figure S61. The 2D charge difference for a slice along the plane passing through O-La-O in unanchored La-NCM (a)

and DA-NCM (b).

Figure S62. The wavelet-transformed EXAFS of cycled P-NCM (a, b) and DA-NCM (c, d).

Figure S63. Comparison of cycling performance between DS-NCM subjected to a 500 °C secondary annealing process and DA-NCM (a) and between DA-NCM and LaMO-NCM (b) at different temperatures.

Figure S64. a. XRD patterns of P-NCA and DA-NCA. b. XRD patterns of pure La_2_Mo_2_O_9_ under the same optimized sintering mechanism as DA-NCA.

Figure S65. High-voltage cycling performance of NCA and DA-NCA under unoptimized (a) and optimized (b) sintering conditions.

Figure S66. XRD pattern of a pure LaMO sample synthesized at 700 °C for 10 hours.

Figure S67. XRD patterns of P-NCM, P-NCM-500 and P-NCM-700.

Figure S68. HRTEM image and SAED patterns of P-NCM-700.

Figure S69. Nyquist plots for P-NCM, P-NCM-500 and P-NCM-700.

Figure S70. Charge-discharge curves of P-NCM-700 at 1 C, 4.5 V under room temperature.

Figure S71. XRD patterns of DA-NCM, DS-NCM-500 and DS-NCM-700.

Figure S72. Nyquist plots for DA-NCM, DS-NCM-500 and DS-NCM-700.

Figure S73. Cycle performance of DS-NCM-500, DS-NCM-700, and DA-NCM at room temperature.

During the two-step modification process, the formation temperature of the anchoring phase LaMO (DS-NCM-500) and the perfect anchoring phase LaMO with complete oxygen vacancy’s formation temperature (DS-NCM-700) were respectively selected for the second annealing^3^. The annealing temperature of 500 °C was selected for the two-step method because it enables the formation of a anchored structure and avoids severe damage to the NCM lattice structure caused by excessively high temperatures or prolonged annealing during secondary modification^4^. Such damage would trigger a significant lattice transformation from the layered to the rock salt phase, leading to a decline in electrochemical performance. Furthermore, for the multi-step method, to prepare pure LaMO with complete oxygen vacancies and form a perfectly anchored structure, an annealing regimen of 700 °C for 10 hours is required (Figure S66)^3^.

However, studies on P-NCM at different temperatures indicate that the annealing regimen at high temperature leads to severe degradation of the P-NCM crystal structure, as shown in Figure S67. Compared to fresh P-NCM and P-NCM annealed at 500 °C (P-NCM-500), due to the oxygen release associated with the phase transition from the layered to the rock salt phase under high-temperature conditions of the sample annealed a second time at 700 °C (P-NCM-700), its lattice undergoes significant contraction, and the (003) peak obviously shifts toward larger angles ^5^. Furthermore, the ratio of the (003) to (104) diffraction peaks decreased significantly, confirming increased cation mixing within the lattice. The HRTEM image and corresponding FFT pattern in Figure S68 also clearly show that a rock salt phase approximately 8 nm thick appeared on the surface of P-NCM after 700 °C annealing, with an interplanar spacing of the (003) plane slightly less than 0.47 nm. The faint diffraction spots in the SAED pattern that do not belong to the layered cathode further confirm that annealing at 700 °C led to severe disorder in the lattice structure. The lattice-structure damage caused by high-temperature annealing reduces the kinetic performance of the Ni-rich cathode (Figure S69) and leads to a decrease in its discharge specific capacity (Figure S70).

Further characterization of the two-step modification system at different temperatures reveals that this intrinsic lattice damage carries over into the DS-NCM system, ultimately resulting in DS-NCM-700 exhibiting the highest degree of cation mixing (Figure S71), the highest charge transfer impedance (Figure S72), and lower discharge specific capacity and capacity retention (Figure S73). Regardless of whether the anchored-structure samples (DS-NCM-500 and DS-NCM-700) were synthesized using the high-temperature or low-temperature two-step method, their performance was inferior to that of DA-NCM, which fully demonstrates the superiority of our design.

Figure S75. (a) Schematic illustrations of anchored oxygen-retardant phase strategy. (b) Comparison of capacity retention between DA-NCM and modified materials from other references.

Table S1. Lattice parameters obtained by XRD Rietveld refinements.

Table S2. Atom coordinates and displacement parameters from XRD refinement results of P-NCM.

Table S3. Atom coordinates and displacement parameters from XRD refinement results of La-NCM.

Table S4. Atom coordinates and displacement parameters from XRD refinement results of DA-NCM.

Table S5. Lattice parameters obtained by NPD Rietveld refinements.

Table S6. Atom coordinates and displacement parameters from NPD refinement results of P-NCM.

Table S7. Atom coordinates and displacement parameters from NPD refinement results of La-NCM.

Table S8. Atom coordinates and displacement parameters from NPD refinement results of DA-NCM.

Table S9. The intensity ratio of (003) and (104) peaks of all the as-prepared samples.

Table S10. Fitting Parameters for the Ni 2p Spectra.^6, 7^

Table S11. Fitting Parameters for the O1s Spectra.^8^

Table S12. BET surface area of the three samples.

Table S13. Comparison in electrochemical performances of DA-NCM under different voltages with the recent similar-focused literatures^6, 9-19^.

Table S14. Mass loading in GITT tests.

Table S15. (003) peak deconvolution fitting results of P-NCM and DA-NCM at 4.0 V and 4.5 V.

Table S16. The intensity ratio of (003) and (104) peaks of the high-rate cycled NCM, La-NCM and DA-NCM at a scan rate of 9° min^−1^.

Table S17. R_ct_ obtained from the in-situ galvanostatic electrochemical impedance spectroscopy test for P-NCM and DA-NCM at different voltages.

References

1.	G. Kresse and A. Ablily, "Efficiency of Ab-initio total energy calculations for metals and semiconductors using a Plane-Wave basis set," *Computation Materials Science* 6 (1996): 15-50.

2.	J. Perdew and A. Ablily, "Atoms, molecules, solids, and surfaces: applications of the generalized gradient approximation for exchange and correlation," *Physical Review B: Condensed Matter* 46 (1992): 6671–6687

3.	Huang Yinglong, He Tianmin, Ji Yuan, Wang Jinxia and W. Deyong., "Preparation and Properties of New Solid Electrolyte La2Mo2O9," *Journal of the Chinese Society of Rare Earths* 22 (2004): 113-118.

4.	B. Zhao, Z. Y. Gu, Y. F. Meng, X. L. Wu and G. Wang, "The Improved Interfacial and Thermal Stability of Nickel‐Rich LiNi0.85Co0.10Mn0.05O2 Cathode in Li‐Ion Battery via Perovskite La4NiLiO8 Coating," *ChemNanoMat* 7 (2021): 672-681.

5.	Z. Cui and A. Manthiram, "Thermal Stability and Outgassing Behaviors of High‐nickel Cathodes in Lithium‐ion Batteries," *Angewandte Chemie International Edition* 62 (2023): e202307243.

6.	M. Yoon, Y. Dong, J. Hwang, J. Sung, H. Cha, K. Ahn, Y. Huang, S. J. Kang, J. Li and J. Cho, "Reactive boride infusion stabilizes Ni-rich cathodes for lithium-ion batteries," *Nature Energy* 6 (2021): 362-371.

7.	S. Alagar, J. Kim, E.-J. Shin, A. Ditter, D. A. Shapiro, N. Kim, W. G. Hong, H. Kim, Y.-S. Yu, G. D. Park and S. M. Jeong, "Correction: Dual-phase modulation via Mo doping and Li3PO4 coating for stabilized LiNi0.9Mn0.07Co0.02Al0.01O2 cathodes in high-energy lithium-ion batteries," *Journal of Materials Chemistry A* 13 (2025): 21087-21087.

8.	H. Sheng, X. H. Meng, D. D. Xiao, M. Fan, W. P. Chen, J. Wan, J. Tang, Y. G. Zou, F. Wang, R. Wen, J. L. Shi and Y. G. Guo, "An Air‐Stable High‐Nickel Cathode with Reinforced Electrochemical Performance Enabled by Convertible Amorphous Li2CO3 Modification," *Advanced Materials* 34 (2022): 2108947.

9.	Z. Dai, Z. Li, R. Chen, F. Wu and L. Li, "Defective oxygen inert phase stabilized high-voltage nickel-rich cathode for high-energy lithium-ion batteries," *Nature Communications* 14 (2023): 8087.

10.	L. Wang, G. Liu, R. Wang, X. Wang, L. Wang, Z. Yao, C. Zhan and J. Lu, "Regulating Surface Oxygen Activity by Perovskite‐Coating‐Stabilized Ultrahigh‐Nickel Layered Oxide Cathodes," *Advanced Materials* 35 (2023): 2209483.

11.	G.-T. Park, B. Namkoong, S.-B. Kim, J. Liu, C. S. Yoon and Y.-K. Sun, "Introducing high-valence elements into cobalt-free layered cathodes for practical lithium-ion batteries," *Nature Energy* 7 (2022): 946-954.

12.	T. Liu, L. Yu, J. Lu, T. Zhou, X. Huang, Z. Cai, A. Dai, J. Gim, Y. Ren, X. Xiao, M. V. Holt, Y. S. Chu, I. Arslan, J. Wen and K. Amine, "Rational design of mechanically robust Ni-rich cathode materials via concentration gradient strategy," *Nature Communications* 12 (2021): 6024.

13.	T. Teng, L. Xiao, L. Shen, J. Ran, G. Xiang, Y. Zhu and H. Chen, "Simultaneous Li2MoO4 coating and Mo6+ doping improves the structural stability and electrochemical properties of nickel-rich LiNi0.83Co0.11Mn0.06O2," *Applied Surface Science* 601 (2022): 154101.

14.	H. Yang, R. M. Gao, X. D. Zhang, J. Y. Liang, X. H. Meng, Z. Y. Lu, F. F. Cao and H. Ye, "Building a Self‐Adaptive Protective Layer on Ni‐Rich Layered Cathodes to Enhance the Cycle Stability of Lithium‐Ion Batteries," *Advanced Materials* 34 (2022): 2204835.

15.	X. Ou, T. Liu, W. Zhong, X. Fan, X. Guo, X. Huang, L. Cao, J. Hu, B. Zhang, Y. S. Chu, G. Hu, Z. Lin, M. Dahbi, J. Alami, K. Amine, C. Yang and J. Lu, "Enabling high energy lithium metal batteries via single-crystal Ni-rich cathode material co-doping strategy," *Nature Communications* 13 (2022): 2319.

16.	H.-H. Ryu, H.-W. Lim, S. G. Lee and Y.-K. Sun, "Near-surface reconstruction in Ni-rich layered cathodes for high-performance lithium-ion batteries," *Nature Energy* 9 (2023): 47-56.

17.	X. Zhou, F. Zhang, X. Fu, N. Zhang, J. Huang, X. Cai, H. Ding, B. Li, L. Niu and S. Li, "Utilizing fast ion conductor for single-crystal Ni-rich cathodes to achieve dual-functional modification of conductor network constructing and near-surface doping," *Energy Storage Materials* 52 (2022): 19-28.

18.	C. Liu, Z. Yi, J. Wan, C. Miao, Z. Wang, J. Wang and W. Xiao, "Nb5+ ions doping and Li3PO4 artificial layer synergetic strategy to strengthen structure, stabilize interface, and promote ions migration for high-performance Ni-rich cathode," *Chemical Engineering Journal* 503 (2025): 158633.

19.	J. Zuo, J. Wang, R. Duan, Y. Bai, K. Xu, K. Zhang, J. Wang, K. Zhang, Z. Yang, Z. Yang, M. Li, G. Cao, Q. Jiang, W. Liu, J. Wang, W. Li and X. Li, "Grain binding derived reinforced interfacial mechanical behavior of Ni-rich layered cathode materials," *Nano Energy* 121 (2024): 109214.
